# Eliminating the type I restriction endonuclease from Pseudomonas aeruginosa PAO1 for optimized phage isolation

**DOI:** 10.1099/mic.0.001634

**Published:** 2025-11-21

**Authors:** Ellie J. Tong, Kate A. Bickerton, Alina J. Creber, Steven L. Porter, Ben Temperton

**Affiliations:** 1 Biosciences, Faculty of Health and Life Sciences, University of Exeter, Stocker Road, Exeter EX4 4QD, UK

**Keywords:** anti-phage defence, *hsdR*, phage isolation, phage therapy, *Pseudomonas aeruginosa*, restriction-modification

## Abstract

Phage therapy is a promising treatment for multidrug-resistant bacterial infections. Due to their high host specificity, phages must be matched to the target clinical strains. Efficiently identifying appropriate phages and producing sufficient titres for clinical use requires comprehensive phage libraries and multiple propagation hosts. An idealized system would use a highly promiscuous bacterial host to isolate a broader range of phages and streamline optimized phage production. Anti-phage defences constrain bacterial host promiscuity, such as restriction-modification systems that recognize and cleave foreign DNA. Here, the type I restriction endonuclease, HsdR, was deleted from *Pseudomonas aeruginosa* PAO1 to make a more promiscuous phage isolation and propagation host. Removal of this endonuclease more than doubled the efficiency of phage propagation on solid media, improved yields from hard-to-propagate phages in liquid bulk-ups and yielded seven times more phages from freshwater samples than wild-type PAO1 – an important step in producing an optimized *P. aeruginosa* strain for isolating and propagating phages for clinical phage therapy.

## Graphical Abstract

**[A]** The type I R-M system is comprised of a methyltransferase (one HsdS and two HsdM subunits) and a restriction endonuclease (one HsdS, two HsdM and two HsdR subunits). **[B]** In wildtype PAO1, the restriction endonuclease destroys phage DNA with unmethylated restriction sites, while the methyltransferase methylates host recognition sites to protect self-DNA from restriction. **[C]** ∆hsdR is optimised for phage isolation and propagation as it lacks the restriction endonuclease, preventing phage DNA cleavage and facilitating efficient phage proliferation. The methyltransferase remains active, so both host and phage DNA are methylated, protecting phage progeny from R-M systems in future hosts. (Created in BioRender.com)

## Data Availability

Sanger sequence data for the ∆*hsdR* deletion mutant is provided at https://github.com/citizenphage/Tong-hsdR. Source data and information on the phages used in this study are provided in the supplementary materials. National Center for Biotechnology accession numbers for phages used in this study are provided in Table S4.

## Code Availability

The data analysis in this study was performed with R and Python code, available here (https://github.com/citizenphage/Tong-hsdR).

## Introduction

*Pseudomonas aeruginosa* is the fourth leading cause of hospital-acquired infections, with high morbidity and mortality in immunocompromised patients with burn wounds, sepsis, traumas and respiratory conditions, including chronic obstructive pulmonary disease and cystic fibrosis [1,2]. The World Health Organization considers *P. aeruginosa* a critical priority for new antibiotics, with 50% of intensive care isolates displaying multidrug resistance [3,4]. The stalled development of novel antibiotics means alternative treatment strategies are required [5].

Phage therapy represents a promising solution, where infections are treated with lytic bacteriophages (phages), the natural predators of bacteria [6]. Phages are highly specific, allowing targeted killing of the pathogen without damaging commensal microbes [7]. However, this specificity also requires phages to be matched to clinical isolates [8]. Comprehensive libraries of characterized phages enable efficient selection of specific phages for patients [9]. Building sufficiently broad phage libraries to match circulating pathogenic strains requires multiple and/or promiscuous bacterial hosts as bait to enrich a diverse range of phages from water samples [10,11]. Additionally, phages must be produced to sufficiently high titres for downstream characterization procedures and clinical use [12,13]. As the phage host range narrows, more hosts are required to propagate and biobank a broad range of phages to high titres, and optimization of host growth parameters suffers from combinatorial expansion. Therefore, phage isolation and production efficiency depend on bacterial host promiscuity, which is constrained by anti-phage defence systems [14,15].

Type I restriction-modification (R-M) systems are the second most common *P. aeruginosa* anti-phage defence system, present in 49% of genomes [16]. R-M systems discriminate between foreign and self-DNA based on methylation status, cleaving phage DNA while protecting the host [17]. Type I R-M systems consist of three subunits: HsdS, HsdM and HsdR [18]. One HsdS and two HsdM subunits form a methyltransferase, which methylates the complementary strand at hemi-methylated recognition sites, protecting host DNA from restriction. Adding two HsdR subunits to the methyltransferase forms a restriction endonuclease, which binds to unmethylated recognition sites in foreign DNA and acts as an ATP-dependent motor, translocating DNA and forming a double-stranded break several thousand base pairs away from the recognition site [19]. Type I R-M systems significantly reduce phage proliferation efficiency [20], thereby limiting host promiscuity.

Phages can escape restriction by methylating their recognition sites, either by using the host or their own methyltransferase, to produce methylated progeny that are protected in future hosts [21,22]. Additionally, methylation status can affect gene expression, influencing phage virulence, packaging and protein expression [23,24]. Therefore, while the restriction endonuclease inhibits phage proliferation, the methyltransferase can facilitate the production of methylated phages that are resistant to recognition and cleavage [21].

Previously, a *Streptococcus pneumoniae* strain lacking type I R-M systems was used as a permissive host to isolate *Streptococcus* phages [20]. One type I R-M system has been identified in the *P. aeruginosa* laboratory strain PAO1, which is routinely used as a phage enrichment host [25,26]. Here, we sought to construct a more promiscuous host for *Pseudomonas* phage isolation by deleting *hsdR* from PAO1, eliminating the restriction endonuclease while maintaining the methyltransferase. We then evaluated whether using ∆*hsdR* as the bacterial host improved the efficiency of phage propagation and isolation from freshwater and wastewater samples.

## Methods

### Constructing ∆*hsdR*

An unmarked deletion of *hsdR* (*PA2732*) was generated by two-step allelic exchange with a suicide vector [27] (strains, plasmids and primers listed in (Tables1^3^). Approximately 500 bp sequences flanking *hsdR* were amplified, joined together by overlap extension PCR and ligated into the plasmid pEX19gm. Modified plasmids were cloned into *Escherichia coli* DH5α and transformed into *E. coli* Rho3 for conjugation into PAO1 on SOB agar (1.5% bacteriological agar, 2% w/v bacto-tryptone, 0.5 % w/v bacto-yeast extract, 0.5 mM NaCl, 2.5 mM KCl and 10 mM MgCl_2_). Double recombinants were obtained by counter-selective plating on Luria Broth (LB) agar containing 100 µg ml^−1^ gentamycin and 5% sucrose. The 1,000 bp region surrounding *hsdR* was PCR amplified and Sanger sequenced to confirm the mutation.

**Table 1.** The strains used in the study

Strain	Description	Source
*P. aeruginosa* PAO1	Wild-type *P. aeruginosa* PAO1	University of Exeter, provided by Angus Buckling
*P. aeruginosa* PAO1 ∆*hsdR*	PAO1 with an unmarked deletion of *hsdR*	This study
*E. coli* DH5α	*E. coli* strain used for plasmid cloning	New England Biolabs
*E. coli* Rho3	*E. coli* strain used for plasmid conjugation	Lopez *et al.* (2009) [54]

**Table 2.** The plasmids used to generate the unmarked deletion of *hsdR*

Plasmid	Description	Accession no.	Source
pEX19gm	Allelic exchange vector encoding gentamycin resistance (*aaC1*) and sucrose sensitivity (*sacB*)	KM887142	Hoang *et al.* (1998) [55]
pEX19gm-∆*hsdR*	Modified pEX19gm containing ~500 bp regions that flank *hsdR*		This study

**Table 3.** The primers used to generate the unmarked deletion of *hsdR*

Primer	Sequence	Description
PA2732P1	CGCGGATCCACAAGCTGCACAGGGCCATC	Forward primer for the amplification of the *hsdR* upstream region and for the overlap extension PCR
PA2732P2	CTCTGTGCATCTTGAAGCGCCTTCTCGCTGGTATCGGTGG	Reverse primer for the amplification of the *hsdR* upstream region
PA2732P3	CCACCGATACCAGCGAGAAGGCGCTTCAAGATGCACAGAG	Forward primer for the amplification of the *hsdR* downstream region
PA2732P4	ACACAAGCTTTTGATACGCGGGAAGATCGG	Reverse primer for the amplification of the *hsdR* downstream region and for the overlap extension PCR
PA2732checkF	CGAGCAAGTTCTACCAGAAG	Forward primer for the check PCR, positioned ~500 bp upstream of *hsdR*
PA2732checkR	CCTGTGATCGCTGACATAAG	Reverse primer for the check PCR, positioned ~500 bp downstream of *hsdR*

### Evaluation of fitness defects via growth curves

Growth curves were generated to determine whether deleting *hsdR* caused fitness defects in the absence of phage. Cultures of ∆*hsdR* and wild-type PAO1 were grown from single colonies in 10 ml of LB (37 °C, 200 r.p.m.) for 3 h and then diluted to a starting OD_600_ of 0.1. One hundred microlitres of each culture was added to six replicate wells of a 96-well plate, and optical density was recorded every 15 min for 16 h in a plate reader (Tecan Sunrise). The intrinsic growth rate (r), carrying capacity (K) and doubling time (DT) were calculated using Growthcurver (v0.3.1) [28].

### Evaluation of promiscuity via spot assays

The ability of 95 *P*. *aeruginosa* phages from the Citizen Phage Library (CPL) [10] to propagate on ∆*hsdR* and wild-type PAO1 on solid media was determined via spot assays [29]. ∆*hsdR* and wild-type PAO1 cultures were grown from single colonies in 10 ml of LB (37 °C, 200 r.p.m.) until mid-logarithmic phase (OD_600_=0.6). Two millilitres of culture was mixed with 6 ml of LB top agar (2.5% w/v LB, 0.65% w/v bacteriological agar, 10 mM MgCl_2_ and 10 mM CaCl_2_) and poured over a 60 ml LB bottom agar plate (2.5% w/v LB, 1% w/v bacteriological agar, 10 mM MgCl_2_ and 10 mM CaCl_2_). Phage lysate was serially diluted with LB to a maximum dilution of 10^−6^. A 5 µl spot of each dilution was spotted onto the top agar and incubated overnight at 37 °C. The efficiency of plating (EoP) was calculated for each phage as the proportion of p.f.u. per millilitre between ∆*hsdR* and wild-type PAO1 [30].

### Evaluation of promiscuity via phage bulk-ups

The efficiency of ∆*hsdR* and wild-type PAO1 as phage propagation hosts was compared by bulking up 17 phages on each strain and comparing the phage titre produced with spot assays on ∆*hsdR* and wild-type PAO1 lawns. Fifty microlitres of phage lysate and 500 µl of overnight bacterial host culture were added to a Falcon tube containing 20 ml of LB, supplemented with 10 mM MgCl_2_ and 10 mM CaCl_2_ and incubated overnight at 37 °C[10]. The cultures were centrifuged at 10,000×***g*** for 15 min at 4 °C, and the supernatant was passed through a 0.22 µm syringe filter to remove bacterial debris. The filtered phage lysates were serially diluted with LB in triplicate to a maximum dilution of 10^−10^. ∆*hsdR* and wild-type PAO1 lawns were set up for spot assays, as before, and 2.5 µl of each dilution was spotted out onto both hosts. Following overnight incubation at 37 °C, plaques were enumerated and the p.f.u. calculated for each phage bulk-up.

### Evaluation of promiscuity via phage isolation

∆*hsdR* and wild-type PAO1 were used as enrichment hosts for 190 freshwater and 95 wastewater samples. Environmental samples were provided to the CPL by citizen scientists and stored in glass amber vials at 4 °C. Wastewater samples were provided by the Environment Agency and stored in 100 µl aliquots at −20 °C. Following the CPL workflow, reported previously [10], samples were enriched by two rounds of incubation with the bacterial host, followed by 0.22 µm filtration. Two microlitres of each enriched sample was spotted onto a host bacterial lawn, incubated overnight at 37 °C and examined for zones of lysis.

### Phage DNA extraction, sequencing and analysis

Agar cores were taken from zones of lysis and transferred to 100 µl of SM buffer (50 mM Tris-HCl (pH 7.5), 0.1 M NaCl and 8 mM MgSO_4_). Fifty microlitres of SM buffer containing phage and 500 µl of overnight bacterial host culture were added to two Falcon tubes containing 20 ml of LB, supplemented with 10 mM MgCl_2_ and 10 mM CaCl_2_ and incubated overnight at 37 °C^10^. The two 20 ml cultures were combined and centrifuged at 10,000×***g*** for 30 min at 4 °C. Thirty millilitres of supernatant was passed through a 0.22 µm syringe filter and treated with 5 µg ml^−1^ DNase I (Roche, Merck, Darmstadt, Germany) and 10 µg ml^−1^ RNase A (Invitrogen, Thermo Fisher Scientific, Waltham, MA, USA) for 30 min at 37 °C. Final concentrations of 10% w/v polyethylene glycol 8000 (PEG8000) and 1 M NaCl were added, mixed by inversion until dissolved and incubated overnight at 4 °C. Precipitated phages were pelleted by centrifugation at 10,000×***g*** for 30 min and resuspended in 1 ml SM buffer. DNA was extracted using the Norgen Phage DNA Isolation Kit (Norgen Biotek, Thorold, ON, Canada) and sequenced at the University of Exeter using 2×150 bp paired-end reads on an Illumina NovaSeq SP platform.

Phage genomes were assembled and annotated as follows (full pipelines are available here: https://github.com/citizenphage/protocols/tree/main/SOPs/Assembly-and-annotation): Reads were QCed with FASTP (v. 0.24.1) with the following settings (--dedup --dup_calc_accuracy 6 --length_required 30 –correction), and high-quality reads were mapped against the genome of the propagation host with minimap2 (v. 2.26) to remove any residual host DNA. Unmapped reads were subsampled to 500-fold coverage with shovill (v. 1.1.0) and assembled with unicycler (v. 0.5.0). Assembly graphs were manually inspected to confirm single circular genomes, with branches of up to 3 bp manually resolved by selection of the most abundant node. Annotation of phage genomes was performed with pharokka (v. 1.7.1) followed by phold (v. 0.1.3).

To quantify the number of HsdR restriction sites in each phage genome, genomic sequences were screened in ipython for non-overlapping recognition sites using the regular expression: ‘GATC.{6}GTC’.

### Statistical methods

Welch two-sample t-tests were used to determine whether the mean growth rate, carrying capacity and doubling time differed significantly between ∆*hsdR* and wild-type PAO1 growth curves. Welch two-sample t-tests were also used to determine whether the mean phage titre (p.f.u. ml^−1^) differed between bulk-ups on ∆*hsdR* and wild-type PAO1. Two-tailed Fisher’s exact tests were used to determine whether the proportion of samples that yield phages differs significantly on ∆*hsdR* and wild-type PAO1.

## Results

### Deleting *hsdR* posed no fitness cost to PAO1 in the absence of phage

Successful in-frame deletion mutants were identified by a check PCR amplifying the region flanking *hsdR* (Fig. S1, available in the online Supplementary Material). Sanger sequencing of the PCR product confirmed the deletion (sequence data is available at: https://github.com/citizenphage/Tong-hsdR). Growth curves showed that ∆*hsdR* incurred no fitness defects in the absence of phage (Fig. S2); there was no significant difference in intrinsic growth rate (r), carrying capacity (K) or doubling time (DT) between ∆*hsdR* and wild-type PAO1 (Table S1).

### ∆hsdR increased the efficiency of phage proliferation on solid media

**Fig. 1.**
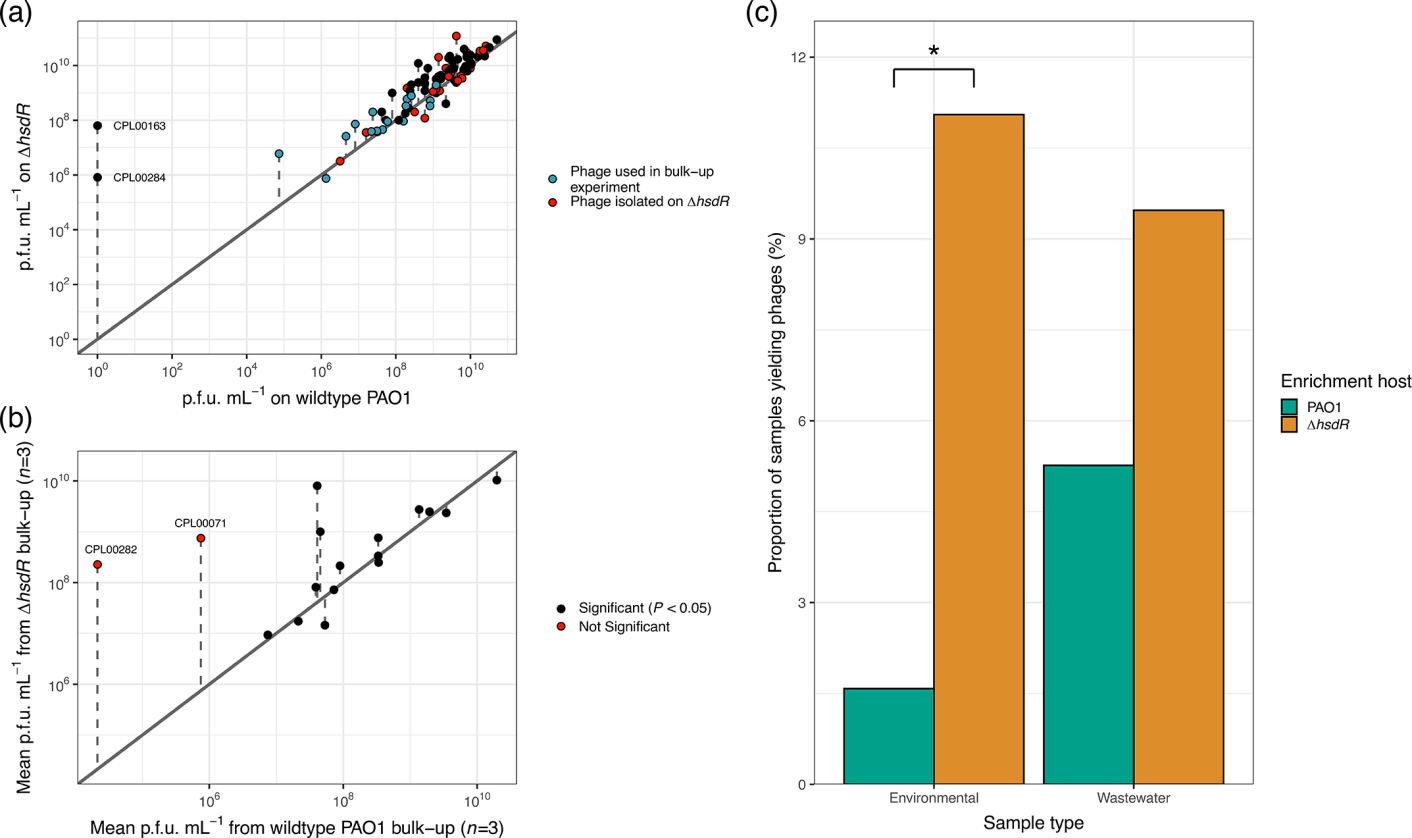
(a) Comparison of concentration (p.f.u. ml^−1^) of 95 phages on wild-type PAO1 against its concentration on the ∆*hsdR* mutant. The distance (dashed line) between each data point and the identity *y*=*x* (solid line) represents the difference in p.f.u. ml^−1^ between the strains. For the phage that could not infect wild-type PAO1, 0 p.f.u. ml^−1^ was converted to 1 p.f.u. ml^−1^ so that they could be represented on the log scale. Points highlighted in red represent phages that were isolated on an ∆*hsdR* mutant; all other phages were isolated on either wild-type PAO1 or a clinical isolate. Points highlighted in blue represent phages that were carried forward for the bulk-up experiment. (b) Comparison of the titre (p.f.u. ml^−1^) of 17 phages produced from bulk-ups on wild-type PAO1 and the ∆*hsdR* mutant, assessed through a spot assay on a ∆*hsdR* overlay plate. CPL00282 and CPL00071 displayed a significantly higher titre on ∆*hsdR* than wild-type PAO1 (Welch’s two-sample t-test, red: *P*<0.05). (c) The proportion of environmental and wastewater samples that yielded phages when enriched using wild-type PAO1 (green) and ∆*hsdR* (orange) as hosts. A significantly greater proportion of environmental water samples yielded phages when enriched on ∆*hsdR* than wild-type PAO1 (two-tailed Fisher’s exact test, *P<0.001).

Spot assays revealed that the mean phage titre of 95 *P*. *aeruginosa* phages was 2.12-fold higher on ∆*hsdR* than wild-type PAO1, with a mean EoP of 4.10 (Fig. 1a). Two phages, CPL00163 and CPL00284, did not form plaques on wild-type PAO1 but did on ∆*hsdR,* with a titre of 6.40×10^7^ and 8.20×10^5^ p.f.u. ml^−1^, respectively, indicating that HsdR-mediated restriction completely inhibits their propagation. The number of HsdR recognition sites (GATC(N)_6_GTC) [26] found within CPL00163 (7) and CPL00284 (6) sat within the range of the number of sites found in 16 other phages that could infect both ∆*hsdR* and wild-type PAO1 (3–7, mean=4.6±0.62 95% CI), suggesting that the prevalence of recognition sites does not determine phage specificity between ∆*hsdR* and wild-type PAO1.

### ∆*hsdR* increased the bulk-up yields of hard-to-propagate phages

Using ∆*hsdR* as a propagation host significantly increased the titre yielded for two hard-to-propagate phages, CPL00071 and CPL00282. When assessed through a spot assay on ∆*hsdR*, the mean phage titre increased from 7.47×10^5^ and 2.13×10^4^ following a bulk up on wild-type PAO1 to 7.47×10^8^ and 2.27×10^8^ following a bulk up on ∆*hsdR*, for CPL00071 and CPL00282, respectively (Fig. 1b, *P*<0.05, *n*=3). Bulking up on ∆*hsdR* did not significantly affect the titres of the 15 other phages tested, which already yielded higher titres from wild-type PAO1 bulk-ups, ranging from 7.47×10^6^ to 1.97×10^10^ (Fig. S4). When bulk-up titres were assessed through a spot assay on wild-type PAO1, significant increases in titre yielded from ∆*hsdR* bulk-ups were observed for CPL00072 in addition to CPL00071 and CPL00282 (Fig. S3). No phages exhibited a significant decrease in titre after bulking up on *∆hsdR* compared to wild-type PAO1 when assessed through spot assays on ∆*hsdR* or wild-type PAO1*.*

### ∆*hsdR* increased the phage isolation yield from freshwater samples

In total, 24 and 14 phages were isolated from 190 freshwater and 95 wastewater samples, respectively. There was a significant 7.00-fold increase in the proportion of freshwater samples that yielded phage on ∆*hsdR* compared to wild-type PAO1 [Fig. 1c, Fisher’s exact test, *P<*0.001, OR=0.130, 95% CI=(0.0243, 0.446)]. Curiously, this was not observed with wastewater samples [Tables S2 and S3, Fisher’s exact test, *P=*0.406*,* OR=0.533, 95% CI=(0.135, 1.854)]. Only 1 of the 13 wastewater samples and 1 of the 23 freshwater samples that yielded phages did so on both ∆*hsdR* and wild-type PAO1, indicating that *Pseudomonas* phage abundance and diversity within and between sample types were highly variable.

## Discussion

Effectively providing phages for clinical use requires comprehensive libraries for rapidly identifying specific phages and streamlined methods for producing sufficiently high titres [9,10, 12]. The efficiency of constructing diverse libraries and propagating a broad range of phages depends on the promiscuity of the bacterial host. Here, we created a more promiscuous *P. aeruginosa* host by deleting the defensive restriction endonuclease HsdR.

Phage propagation was 2.12-fold more efficient on ∆*hsdR* than wild-type PAO1 on solid media, aligning with previous findings that restriction-modification systems significantly limit phage proliferation [20,21]. One study reported a 10,000-fold decrease in phage SpSL1 proliferation when the phase-variable type I R-M system SpnIV was expressed in *S. pneumoniae* [20]*.* Here, we found that deleting the HsdR subunit alone was sufficient to increase phage proliferation. This deletion eliminates phage DNA cleavage by the restriction endonuclease but maintains the methyltransferase, such that phage progeny may still be methylated and protected in future hosts [21,22]. Similarly, a previous study found that increasing the ratio of methyltransferases to restriction endonucleases in an *E. coli* type II R-M system abolished protection against phage [21].

Two phages (CPL00163 and CPL00284) could infect ∆*hsdR* but not wild-type PAO1. Thus, ∆*hsdR* permits infection by some phages that are otherwise completely inhibited by restriction. CPL00163 and CPL00284 possessed a similar number of recognition sites to other phages that can infect wild-type PAO1, indicating that a high frequency of recognition sites is not the reason that these phages are particularly sensitive to restriction [31]. Alternatively, they might lack anti-restriction strategies that other phages possess [32,33]. For instance, some phages avoid recognition by acquiring methylated or non-canonical bases in their recognition sites [24,34, 35] or by encoding anti-restriction proteins like Ocr, which blocks the endonuclease binding groove [36].

*∆hsdR* also significantly increased the titre yielded from bulk-ups of CPL00071 and CPL00282, two phages that yield low titres (<10^6^ p.f.u. ml^−1^) from wild-type PAO1 bulk-ups. Bulking up on *∆hsdR* compared to wild-type PAO1 did not significantly affect the titre of the other 15 phages tested*,* despite most of these phages proliferating more efficiently on *∆hsdR* on solid media. Other factors may be preventing phage titre from increasing, such as host growth rate, influenced by oxygen or nutrient availability, or phage–host dynamics, including the development of phage-resistant mutants [37]. Importantly, no phages exhibited a significant decrease in titre from bulking-up on *∆hsdR* compared to wild-type PAO1. Thus, *∆hsdR* could serve as a promiscuous host to streamline phage production by permitting the propagation of phages that are unable to infect wild-type PAO1 or produce insufficient titres on wild-type PAO1, without negatively impacting the propagation of other phages.

Using ∆*hsdR* as the enrichment host increased freshwater enrichment yields sevenfold but had no significant impact on wastewater enrichment yield. Phage concentrations are typically 10–1000 times higher in wastewater than in freshwater [38,40], making wastewater phage enrichments generally more successful [41]. Since ∆*hsdR* enables more efficient phage proliferation than wild-type PAO1, using ∆*hsdR* as the host may increase enrichment yields by improving the chance of recovering low-abundance phages. As a result, eliminating *hsdR* may have a more significant impact on freshwater isolation yields, where phages are typically present at lower concentrations.

The overall success rate of phage enrichment was relatively low compared to previous studies [11,42, 43]. One study achieved a 79.4% success rate for isolating *P. aeruginosa* phages from 20 to 30 ml samples of unprocessed wastewater [42]. We may have recovered fewer phages from wastewater as our samples were stored at –20 °C and freeze-thawed, potentially damaging phages [44,45]. Additionally, enriching smaller 1 ml samples may have reduced the success rate per sample but allowed overall higher throughput. The relatively low chance of yielding a phage from each sample with this high-throughput method may explain why there was little overlap in the samples that yielded phage on ∆*hsdR* and wild-type PAO1, as it was unlikely for the same phage to be isolated more than once.

*∆hsdR* facilitated the proliferation of four hard-to-propagate phages, streamlining phage production by reducing the need for specific, optimized hosts for hard-to-propagate phages. To further improve ∆*hsdR* as a propagation host, secreted virulence factors, such as exotoxin A, could be deleted to expedite downstream purification [46,47]. As freshwater isolation yields were seven times greater on ∆*hsdR*, a broader range of phages could be efficiently obtained for phage libraries using this strain. The recent discovery of phage defence islands in bacterial genomes has uncovered dozens of novel anti-phage defence mechanisms [15,32, 48], which could be future targets for deletion to further increase ∆*hsdR* promiscuity. Beyond defence systems, host promiscuity is constrained by phage receptor specificity [49,50]. Similar deletions of anti-phage defence systems and toxins could be made in other *P. aeruginosa* strains that possess different variations of common phage receptors, such as the LPS and type IV pili [51,53]. Ultimately, this would create a panel of characterized, promiscuous and safe hosts for the streamlined isolation and propagation of a diverse range of phages.

## Supplementary material

10.1099/mic.0.001634 Uncited Supplementary Material 1.

10.1099/mic.0.001634 Uncited Supplementary Material 2.

## Abbreviations

CPL: Citizen Phage Library
EoP: efficiency of plating
R-M: restriction-modification
