# Supplementary material for: Eliminating the type I restriction endonuclease from Pseudomonas aeruginosa PAO1 for optimized phage isolation
Source: Microbiology (Reading). 2025 Nov 21;171(11):001634. doi: 10.1099/mic.0.001634 (PMC12638068; doi:10.1099/mic.0.001634)
Supplement: Uncited Supplementary Material 1. [file mic-171-01634-s001.pdf]

Supplementary materials

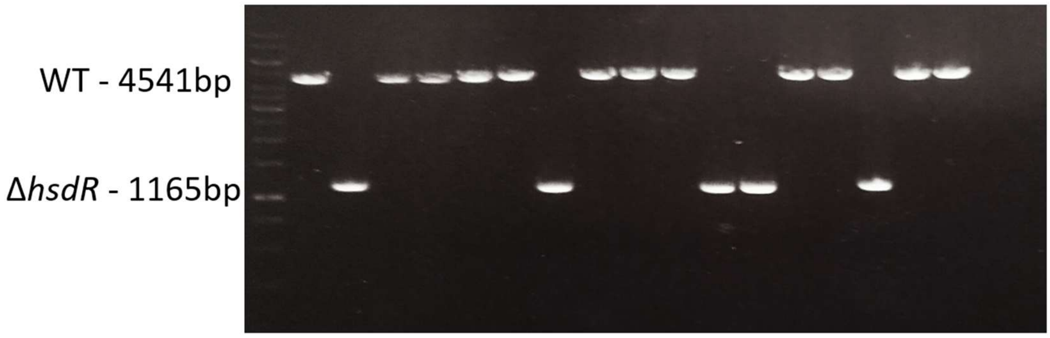

Supplementary Figure 1: An image of the check PCR products run on a 1% agarose gel. The sequence flanking *hsdR* was PCR amplified to distinguish successful in-frame deletion mutants (1165 bp) from wildtype (WT) revertants (4541 bp).

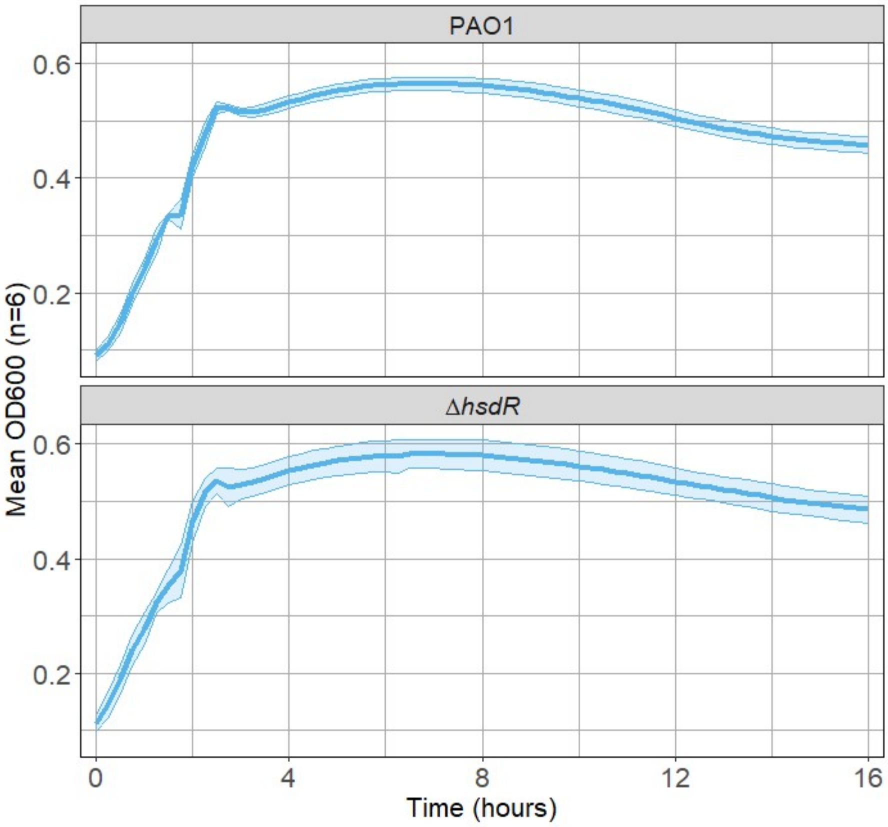

Supplementary Figure 2: The mean OD_{600} of Δ*hsdR* and wildtype PAO1 over 16 hours at 37 °C. Ribbons represent 95% confidence intervals around the mean.

	Carrying capacity (K)	Intrinsic growth rate (r)	Doubling time (DT)
**Mean value for PAO1**	0.394	2.51	0.277
**Mean value for Δ*hsdR***	0.396	2.42	0.287
**Degrees of freedom (df)**	6.77	6.48	6.17
***p*-value**	0.822	0.242	0.233

Supplementary Table 1: The mean carrying capacity (K), intrinsic growth rate (r) and doubling time (DT) of PAO1 and Δ*hsdR* determined using Growthcurver[28]. Welch two sample t-tests were used to determine if the mean growth parameters differed significantly between PAO1 and Δ*hsdR*.

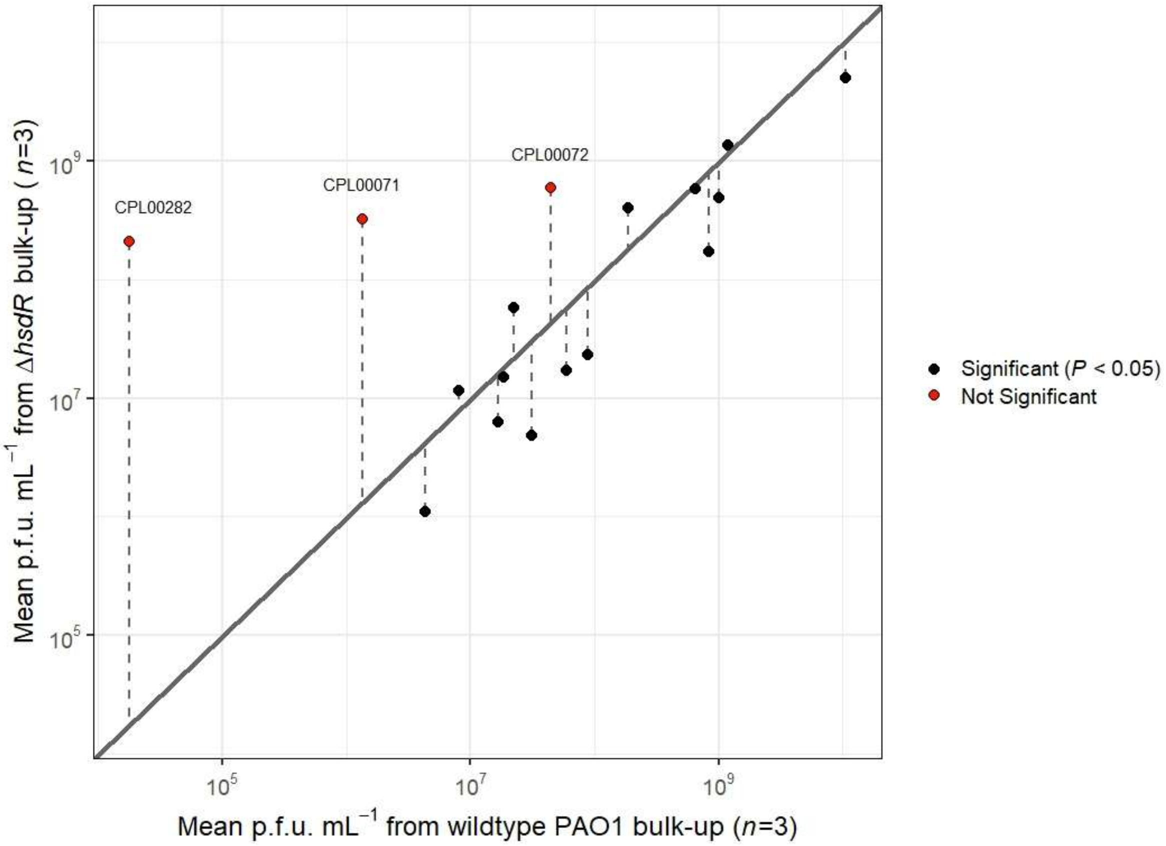

Supplementary Figure 3: Comparison of the titre (p.f.u. mL^{-1}) of 17 phages produced from bulk-ups on wildtype PAO1 and the Δ*hsdR* mutant, assessed through a spot assay on a wildtype PAO1 overlay plate. CPL00282, CPL00071 and CPL00072 displayed a significantly higher titre on Δ*hsdR* than wildtype PAO1 (Welch's two-sample T-test, red: *p*<0.05). The p.f.u. mL^{-1} of the 14 remaining phages was not significantly affected by bulking up on Δ*hsdR* (black).

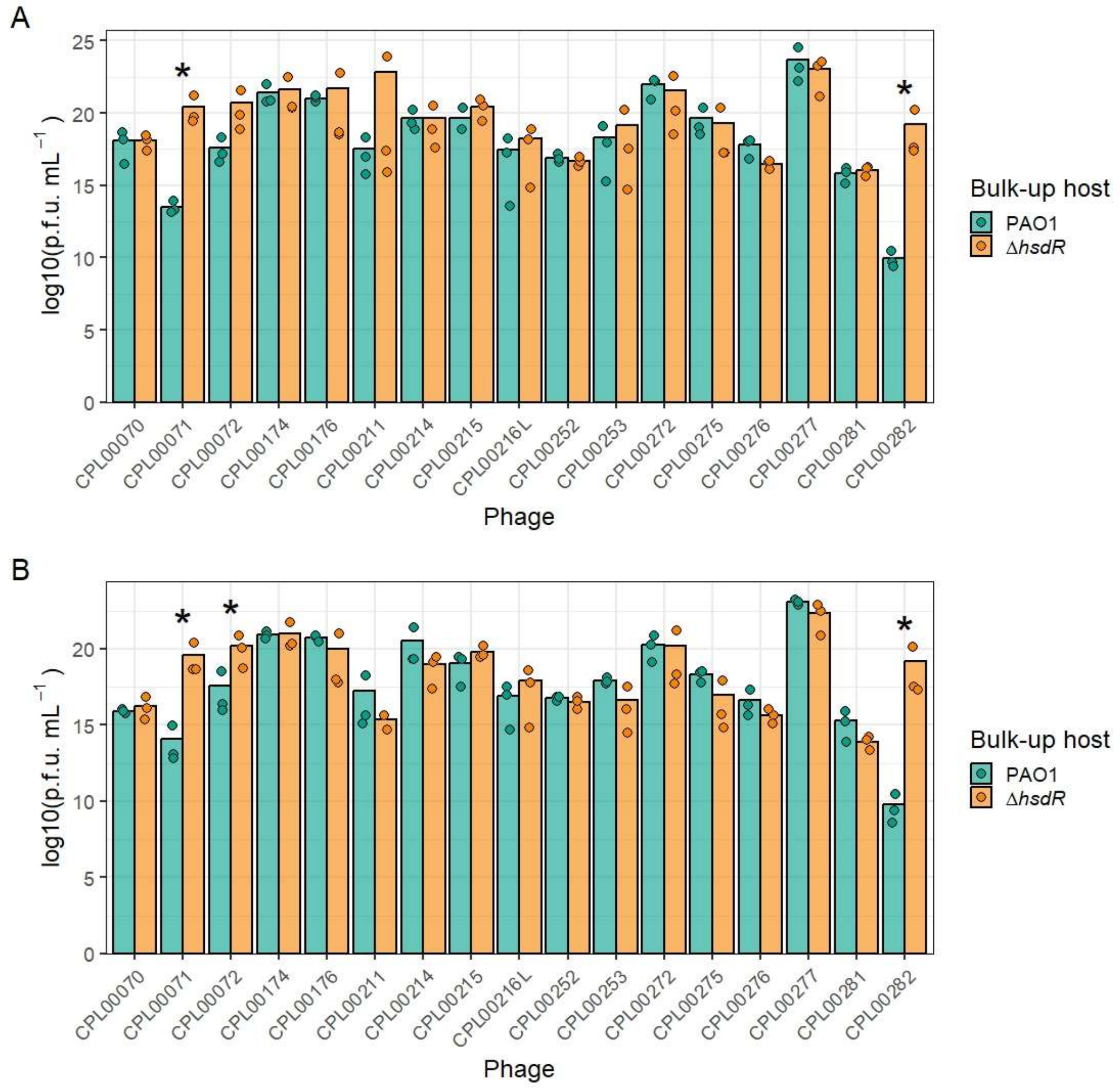

Supplementary Figure 4: Bar graphs showing the individual repeats of phage p.f.u. after being bulked up on either PAO1 (green) or Δ*hsdR* (orange) and spotted out onto **[A]** Δ*hsdR* or **[B]** wildtype PAO1 for plaque enumeration and p.f.u. calculation.

	**Zone of lysis**	**No zone of lysis**
**PAO1**	3	187
**Δ*hsdR***	21	169

Supplementary Table 2: Contingency table for the proportions of freshwater samples that yield phage on PAO1 and Δ*hsdR*

	**Zone of lysis**	**No zone of lysis**
**PAO1**	5	90
**Δ*hsdR***	9	86

Supplementary Table 3: Contingency table for the proportions of wastewater samples that yield phage on PAO1 and Δ*hsdR*
